# Human-Wildlife Conflicts in the Southern Yungas: What Role do Raptors Play for Local Settlers?

**DOI:** 10.3390/ani11051428

**Published:** 2021-05-17

**Authors:** Amira Salom, María Eugenia Suárez, Cecilia Andrea Destefano, Joaquín Cereghetti, Félix Hernán Vargas, Juan Manuel Grande

**Affiliations:** 1 Laboratorio de Ecología y Conservación de Vida Silvestre, Centro Austral de Investigaciones Científicas (CADIC-CONICET), Bernardo Houssay 200, Ushuaia 9410, Argentina; 2 Departamento de Ecología, Genética y Evolución, Facultad de Ciencias Exactas y Naturales, Universidad de Buenos Aires, Intendente Güiraldes 2160, Buenos Aires C1428EGA, Argentina; 3 The Peregrine Fund, 5668 West Flying Hawk Lane, Boise, ID 83709, USA; hvargas@peregrinefund.org (F.H.V.); manuhola@yahoo.es (J.M.G.); 4 Grupo de Etnobiología, Departamento de Biodiversidad y Biología Experimental, Facultad de Ciencias Exactas y Naturales, e Instituto de Micología y Botánica (INMIBO), Universidad de Buenos Aires, CONICET-UBA, Intendente Güiraldes 2160, Buenos Aires C1428EGA, Argentina; eugesuarez78@gmail.com; 5 Área de Agroecología, Facultad de Agronomía, Universidad de Buenos Aires, Av. San Martín 4453, Buenos Aires C1417, Argentina; cdestefano@agro.uba.ar; 6 Las Jarillas 83, Santa Rosa 6300, Argentina; joaquincereghetti@gmail.com; 7 Colaboratorio de Biodiversidad, Ecología y Conservación (ColBEC), INCITAP (CONICET-UNLPam), Consejo Nacional de Investigaciones Científicas y Técnicas, Facultad de Ciencias Exactas y Naturales (FCEyN), Universidad Nacional de La Pampa (UNLPam), Avda, Uruguay 151, Santa Rosa 6300, Argentina

**Keywords:** human-wildlife conflict, human-wildlife interactions, perceptions, attitudes, andean condor, *Vultur gryphus*, birds of prey, socio-ecological system

## Abstract

**Simple Summary:**

Human-Wildlife conflict (HWC) has become an important threat producing biodiversity loss around the world. As conflictive situations highly depend on their unique socio-ecological context, evaluation of the different aspects of the human dimension of conflicts is crucial to ensure wildlife conservation and people’s well-being at the same time. Raptors have been historically involved in HWC because some of them are considered livestock predators. We conducted interviews to evaluate the existence, extent and characteristics of human-raptor conflicts in the Southern Yungas region in Northwestern Argentina, identified as a priority area for raptor research. Our results suggest that conflict is only concentrated in certain high-profile raptor species—particularly those considered cattle predators—with a general high tolerance towards poultry predators. Livestock and poultry rearing are the only socio-demographic variables associated with the existence of HWC, independently of age, gender and occupation of interviewees. Andean condor, a Vulnerable species persecuted in some regions of Argentina, was the most conflictive species as it is locally considered a livestock predator. Though reports of Condor persecution were scarce in our study, negative attitudes towards this species, together with the increasing number of mass poisonings, highlight the need to address this conflict through true intercultural dialogue and transdisciplinary approaches.

**Abstract:**

Wildlife persecution due to human-wildlife conflict has become a serious concern for biodiversity conservation, especially for many endangered species. In this context, conservation approaches need to consider the socio-ecological dimensions of each particular situation. The aim of this study was to evaluate the existence, extent and social characteristics of Human-Raptor Conflicts (HRC) in the Southern Yungas region in northwestern Argentina. We conducted 115 semi-structured interviews in 21 sites and analyzed attitudes and associations between sociodemographic variables and the existence of HRC. Forty percent of interviewees showed negative attitudes towards raptors, mainly with those species considered livestock predators rather than poultry predators. A total of 11 species were regarded as conflictive because of predation on domestic animals, of which Andean condors showed the highest conflict. The only socio-demographic factor affecting conflicts was livestock and poultry rearing, independently of age, gender and occupation of interviewees. The fact that only 8.7% of interviewees reported taking direct actions towards conflictive species indicates a relatively peaceful coexistence of people with raptors. Nevertheless, negative attitudes towards Andean condor together with their extreme susceptibility to any increase in non-natural mortality indicate the need of an integral conservation approach to tackle future threats for this species’ conservation in the area.

## 1. Introduction

Persecution due to Human-Wildlife Conflict (HWC) has become a serious and widespread concern for biodiversity conservation, as it imposes a serious threat to the survival of many globally endangered species [1]. Although different situations and motivations may be behind conflictive human-wildlife interactions [2], livestock and game predation, as well as human safety, are globally the most common sources of what is known as Human-Wildlife Impacts (HWI) [3,4]. Simultaneously, HWC involves underlying Human-Human Conflicts (HHC) which arise when two or more stakeholders have different wildlife management practices, goals or priorities [3,5]. HHC usually increase when the actions of one stakeholder are perceived to be at the expense of another stakeholder’s interests; as is usually the case between conservation biologists or control agents (such as legislators and park-rangers) and local people, especially gamekeepers or rural settlers [6]. Therefore, understanding the ecological context is not enough for the resolution or management of HWC; as these conflictive situations highly depend on their unique local socio-ecologic contexts [7,8]. The only way to ensure both wildlife conservation and people’s well-being at the same time, is to consider the different aspects of the human dimension of conflicts, including the socio-economic context, concepts, values, perceptions and attitudes of the stakeholders involved, and an evaluation of the interactions between them [5,9,10,11,12]. For this, in the last decades, interdisciplinary fields of study such as ethnozoology, conservation marketing or social psychology, have made relevant contributions to biodiversity conservation, as they provide useful information on social beliefs, practices and rights, as well as methodological tools to reach participatory and consensual solutions that lead to human-wildlife coexistence, which are crucial for conservation programs to be truly successful [2,13,14,15,16]. 

Historically, raptors have been involved in HWC [17,18], and persecution is stated as one of the major threats for these birds in many regions of the world [19,20]. To address these conflicts, certain studies evaluated the economic impacts produced by raptor predation on livestock and suggested that losses of domestic animals by raptor predation are usually exceptional and/or less than losses produced by other natural causes such as diseases [4,21,22]. Nonetheless, financial impact is context-dependent, and therefore can be perceived as low by researchers while actually being high in relation to the income and the vulnerability of the person or families involved, especially when they are highly dependent on rural activities [23,24]. On the other hand, even if monetary losses are indeed insignificant, the differences found between damage perception and actual monetary losses suggest that some intangible costs related with human well-being (e.g., psychological cost of fear or traumatic experiences) could be more important than tangible costs (e.g., monetary losses) [25,26]. For example, negative perceptions and attitudes may be associated with social representations rooted in cultural or traditional views, related with symbolic, meaning-making and emotive realms, that for local settlers could be more important than scientifically-acquired evidence [27,28]. In recent years, several studies have addressed this and other factors of the human dimension of human-raptor conflict and contributed to the comprehension of local perceptions towards raptors in different regions and environments [21,29,30,31,32,33,34,35,36,37,38,39]. 

The Tropics of Central and South America have been identified as one of the highest priority areas for raptor research and conservation [40], and despite remarkable growth in studies over recent years, scientific knowledge of raptors in Argentina remains scarce. There is, however, evidence of population retraction or at least of widespread persecution across the country for some species. This is the case of the Crowned solitary eagle (*Buteogallus coronatus*), the Andean condor (*Vultur gryphus*) and the Striated caracara (*Phalcoboenus australis*), for which persecution led to a decline or extinction of local populations [41,42,43,44]. 

In particular, the Southern Yungas ecoregion in Northwestern Argentina is a hotspot of biodiversity, hosting a high number of predatory and scavenging raptors [45,46], and thus is identified as a priority area for raptor research [47]. Previous studies in other areas of South America have recorded HWC and related it to negative perceptions associated with predation of domestic animals for some of the species that are present in this region; such as the Black-and-chestnut eagle (*Spizaetus isidori*), the Andean condor, the Southern caracara (*Caracara plancus*), the Black-chested buzzard-eagle (*Geranoaetus melanoleucus*), the Crowned solitary eagle, the Black-and-white hawk-eagle (*Spizaetus melanoleucus*) and the Roadside hawk (*Rupornis magnirostris*) [21,22,29,30,34,38,48,49,50,51]. While most of these species are classified as Least Concern [52], some of them have concerning conservation status and thus to identify the existence of potential conflicts could be critical to promote proper conservation measures in the area. The Andean condor is listed in the CITES Appendix I and was recently uplisted to Vulnerable by the IUCN, while the Black-and-chestnut eagle (hereafter BCE) and the Crowned solitary eagle are classified as Endangered worldwide [52]. The BCE is a species that inhabits exclusively the subtropical and tropical montane cloud forests of the Andean slopes [53]; and besides habitat destruction, direct human persecution is inferred to be the main driver of its continuing decline [52]. Although between 1959 and 2020 there were no records of BCE persecution in Argentina [54], (Grande pers.obs.), predation on poultry, the main driver of conflicts involving this species in Colombia and Ecuador [29,30,49], has been recorded in the only nest documented for the species in the country [55]. On the other hand, the Andean condor and the Crowned solitary eagle are directly persecuted in some regions of Argentina as they have been accused of preying on livestock [21,34,41,48]. Given that most local settlers of the Yungas are traditionally seasonal farmers and transhumant herders [56], there could be a potential human-raptor conflict. However, there is no information about these potential conflicts in the rural socio-ecosystems of the Yungas, nor studies about knowledge, perception and attitudes of local settlers towards raptors in general for the area. 

Here, we evaluate the existence, extent and characteristics of human-raptor conflicts in the portion of the Southern Yungas ecoregion in Northwestern Argentina. For this, we analyzed perceptions and attitudes of local settlers towards raptors, we assessed if there was any conflict due to predation of domestic animals involving raptors, and we evaluated the association between sociodemographic variables and the existence of conflicts with raptors and in the context of potential conflicts with other wildlife. We predicted that the occupation of interviewees and the rearing of domestic animals will determine local settlers’ perceptions and attitudes towards raptors and the existence of conflict with these species. We also expected that conflicts will be associated only with those raptors species that are perceived as predators of domestic animals.

## 2. Materials and Methods 

Ethics statement: We conducted research under permits granted by the Secretaría de Ambiente del Ministerio de Ambiente y Producción Sustentable de la Provincia de Salta (Res. 321/16, expte. 0090227-86919/2016- 0) and the Dirección Provincial de Biodiversidad, Secretaría de Gestión Ambiental de Jujuy (Res. 107/DPB-2015). In all cases, prior informed consent was obtained verbally from each participant and community leaders [57]. The data that support the findings of this study are available from the corresponding author upon reasonable request. 

### 2.1. Study Area 

**Figure 1:**
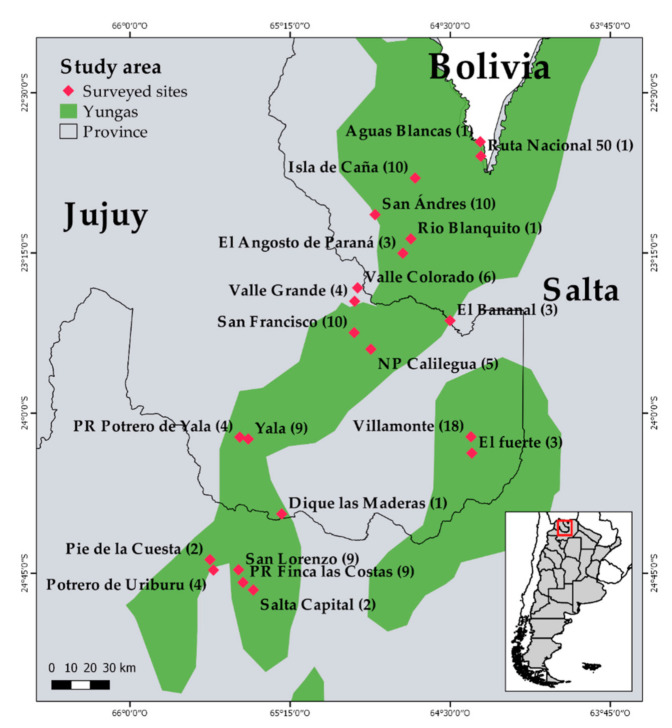
Distribution of the Yungas ecoregion in the study area and sites where interviews were performed. The number of interviews carried out at each settlement is indicated in parenthesis. NP = National Park, PR = Provincial Reserve.

The study was conducted in 21 sites in the provinces of Jujuy and Salta, in Northwestern Argentina. The study sites are in the southern portion of the Southern Yungas’s phytogeographic province (Figure 1). Six sites corresponded to national, provincial and/or private protected areas (Calilegua National Park, Finca las Costas Provincial Park, Potrero de Yala Provincial Park and Ecoportal de Piedra Private Reserve) and four to territories of native communities (Tinkunaku Kolla Community and Finca Santiago).

The Southern Yungas ecoregion occupies the eastern slopes of the Andean mountains in Bolivia and Argentina, and is distinguished by a persistent or partial cloud and fog cover [58], and a subtropical climate with cold and dry winters and warm and rainy summers. The mean annual temperature ranges between 14 °C and 26.5 °C, and mean annual precipitation ranges between 700 mm and 2400 mm, with 80–90% of rainfall concentrated from November to March [59]. The sharp altitudinal gradient produces changes in precipitation and humidity generating four clear vegetation zones: the premontane forest (up to the 500 m a.s.l.), the low montane forest (500–1500 m a.s.l.) and the high montane forest (1500–3000 m a.s.l.), which from now on will be classified under the local name “monte”, and the cloud pastures (2800–3500 m a.s.l.), called “cerro” from now on [60,61]. It is estimated that up to a 40% of the species of fauna and flora from Argentina can be found in the Yungas, which represents less than 2% of the area of the country [62]. For predatory and scavenging raptors, species richness is even higher and up to 78.33% (47/60) of the raptors present in Argentina can be found in the Yungas (Table S1) [63]. Despite its great biodiversity, 31% of the Yungas forest area (14,000 km^2^) has been transformed into productive activities, and the remaining 31,000 km^2^ are seriously threatened by deforestation pressure for the expansion of agriculture, livestock overgrazing and selective logging [62,64].

The human population of this region is a combination of societies with different cultural and historical backgrounds, including mestizos and several indigenous peoples, particularly Kollas and Ava Guaraní [57,65]. The main economic activity in the region is sugar agroindustrial monoculture production centered in lowland areas formerly covered by premontane forest, which exacerbates the economic inequality that characterizes the region. Most of the studied communities still maintain their traditional activities of subsistence, such as extensive cattle ranching, familiar agriculture, poultry rearing and hunting as their main or supplementary source of income. In particular, they are involved in a combination of rotational agriculture and the movement of livestock herds among different altitudinal zones (known as transhumance) in an annual cycle [66]. During the summer livestock herds are taken to the high lands (“cerro”), and lower lands (“monte”) are used during the winter. This deeply characterizes people perceptions and beliefs which are aligned with a “cerro-monte” duality [67].

### 2.2. Field Surveys

During 2016, as part of a broader multidisciplinary research that aimed to study the local knowledge and human-wildlife interactions involving the BCE in particular and diurnal raptors in general [68], we interviewed 115 local settlers older than 18 years of age; 64 in Jujuy and 51 in Salta, respectively. Interviewees were selected by means of the snowball sampling technique [69].

Data were gathered through semi-structured interviews (Supplementary Materials) and collaborative field work with interviewees [69,70]. During the interviews, additional material was used, such as photographs and vocalization records of different raptors present in the study area, including Andean condor, Black-chested buzzard eagle, Black-and-white hawk-eagle, Roadside hawk, Great black hawk (*Buteogallus urubitinga*), Red-backed hawk (*Geranoaetus polyosoma*), Ornate hawk-eagle (*Spizaetus ornatus*), BCE, Harpy eagle (*Harpia harpyja*), Southern caracara and Crowned solitary eagle. Although the latter mainly inhabits open woodlands in xerophytic forests, we included the species in our catalog as there are some records in the Yungas ecoregion [71]. Each interview was organized in three main sections: (i) socio-demographic characteristics of interviewees [i.e., age, gender, ethnic affiliation, occupation, place of residence, time of residence and whether they currently raise or ever have raised livestock (including cattle, horses, sheep, pigs and goats) or poultry]; (ii) interviewees recognition, perceptions and attitudes towards the raptors of the area. Here, we asked interviewees to mention which raptors they knew, to describe the morphological and behavioral characteristics they use to recognize them, to tell us any appreciation, feeling and/or considerations about them, and to point them out among the photographs of the birds; and (iii) existing conflicts between local settlers and wildlife (particularly raptors), asking interviewees which wildlife species they considered to be problematic or conflictive, especially due to predation of domestic animals.

### 2.3. Data Analysis 

We considered people’s “perception” as the thoughts and representations resulting from the process by which people interpret and organize sensations and experience of their world [72]. On the other hand, we defined people’s “attitude” as the interviewees’ evaluation of their perceptions and the resulting feeling or opinion towards different raptor species. We categorized these evaluations *ad hoc*, under an *etic* perspective, as negative, neutral or positive attitudes. Expressions and opinions that related interviewee’s perception of raptors with cultural, ecological, aesthetic, spiritual or touristic value were associated with positive attitudes. On the other hand, negative connotations and lore, angry or fearful expressions and conflict declarations towards raptors were associated with negative attitudes. The absence of any of these signs was considered a neutral attitude. To evaluate perceptions and attitudes, we used a holistic approach, integrating qualitative and quantitative analytical methods, such as content analysis and Chi-Square tests. Fisher’s exact test was used when comparing groups which had less than five respondents. 

To evaluate the existence of social conflict with wildlife, two conflict indices based on indices normally used in ethnobiology that take into account consensus level between interviewees were calculated [73,74,75]. One index (ICo) was estimated for each species to evaluate which ones were considered more conflictive by interviewees and the other one (OC) was estimated for each occupation category to evaluate if conflict with wildlife varies with the interviewees’ occupation:

(a) Index of Conflict (ICo), which consists of the relative frequency of citation, was defined as the number of interviewees that reported a conflict with the species over the total number of interviewees [74]. For Jaguars (*Panthera onca*) we calculated this index including only the interviews done in areas where the species is still present using thus a lower total number of interviewees in comparison to the other species evaluated. Only significant conflict reports (with ICos > 5%) are reported here.

ICo_s_ = n_is_/n * 100,

n_is_ = number of interviewees that manifest conflict with the species s, (1)

n = total number of interviewees.

(b) Conflict Index by Occupation (OCi), where each addend is a measure of the relative importance of each conflictive species. Each index was pondered by the proportion of interviewees in each occupation category.

OCi = (n_ai_+n_bi_+... + n_ni_)/( n_o_),

n_ai_ = number of interviewees that manifest conflict with a,

n_bi_ = number of interviewees that manifest conflict with b, (2)

n_ni_ = number of interviewees that manifest conflict with n,

n = total number of interviewees dedicated to occupation O.

Generalized linear models (GLM) with binomial distribution of errors were used to evaluate the influence of social factors on people’s conflict with wildlife. The social factors used as candidate explanatory variables in the models were gender, age, occupation, ethnic affiliation, place of residence, time of residence, livestock rearing and poultry rearing. The variable “occupation” was categorized for this analysis; “tourist guides”, “logging” and “highway employees” were grouped under the category *outdoor occupation*, while, ”market employees”, “politicians”, “army members”, “school cookers” and “people with administrative jobs” were group under the category *others*. Multicollinearity of the explanatory variables was evaluated using the function vif (for variance inflation factor) of the package “car” for R software [76], and variables with vif > 10 were discarded. The final set of explanatory variables included were: gender, age, occupation, time of residence, livestock rearing and poultry rearing. For each response variable we run the full models.

To assess people’s conflict with wildlife we constructed the response variable as a matrix, including the proportion of species that were considered conflictive by each interviewee over the total number of species that were reported as conflictive. Separate models were fitted to evaluate the influence of these social factors on the conflict expressed for each species considered conflictive with ICos > 5%. To assess the significance of each single explanatory variable the function Anova of the package car was used. A statistical significance was considered when *p* < 0.05.

## 3. Results

### 3.1. Socio-Demography of Surveyed People

The interviewees’ age ranged mostly between 38 and 59 years, and were mostly men (75.65%, Table 1). Most interviewees had jobs strongly related with nature, including livestock production, agriculture and logging (57.39%), and ornithological tourism, private reserve administration, an d park ranging (19.13%). The rest of the interviewees had diverse jobs including tourism, market employees, road maintenance employees and school teachers. Furthermore, most of respondents, and at least half of the interviewees of each occupation category, except for park rangers, reared poultry (67.82%) or livestock (66.96%) at the time of the interview or in the past.

### 3.2. Perceptions and Attitudes toward Raptors

Almost half of the interviewees showed a neutral or indifferent attitude towards raptors (45.22%, 52/115), while 14.78% (17/115) expressed positive attitudes mainly towards eagles, which are perceived as beneficial species or related with aesthetic values (X_2_ = 58.11, gl = 3; *p* < 0.001). This positive view was mostly related with the idea that they feed on snakes. This was considered beneficial by interviewees because, in part, some of them associate snakes with religious or superstitious beliefs with negative connotation (e.g., they are linked to hell), and, at the same time, they are considered dangerous for livestock, poultry and people. The remaining people (40%, 46/115) had negative attitudes towards raptors. In particular, among the interviewees who have rural activities as their main occupation (related with *farming* and *livestock production*), 56.67% (34/60) had negative attitudes towards raptors. This percentage decreased to 32.72% (18/55) (Fisher test, *p* = 0.0358) among the rest of the interviewees, who are engaged in other types of activities. 

On the other hand, although some raptors were considered as poultry predators, few interviewees perceived them as harmful or conflictive species. For instance, only 6 of 21 interviewees who reported that eagles prey on hens showed negative attitudes and perceptions towards this group. This is also true for the Roadside hawk, which was identified as a chicken predator by 57.39% (66/115) of the interviewees but only 25.76% (17/66) of them reported having conflicts with this species. 

At least three raptors or group of raptors species were reported to prey on livestock: Southern caracara and vultures (*Coragyps atratus* and/or *Cathartes* spp., which seem to be one ethnospecies among local people, i.e., they do not distinguish them as different species) were reported to prey on young lambs and piglets, and Andean condors, that were attributed of preying on livestock calves. Although the number of interviewees that reported livestock predation by the former two species was low (5.22%, 8/115), almost all of them (75%, 6/8) expressed having conflicts with these species. On the other hand, half of the interviewees (50.43%, 58/115) considered the Andean condor as a predator and 68.97% (40/58) of them declared having a conflict with the species (X_2_ = 57.3, gl = 1, *p* < 0.001).

### 3.3. Conflicts with Wildlife 

As part of the results, we also obtained information about other species apart from raptors that are involved in HWC due to predation of domestic animals. This information mostly arose when we asked people to mention any wildlife species that they considered problematic or conflictive. 

More than half of the interviewees declared having conflicts with wildlife due to predation of domestic animals (59.13%, 68/115). In 13.91% of the cases (16/115) interviewees exclusively expressed to have conflicts with mammal predators, while 23.48% (27/115) exclusively expressed they had conflict with raptor predators (X_2_ = 6.4, gl = 1, *p* = 0.012). A total of 16 species were reported to prey on domestic animals, but only 11 species were considered conflictive by the interviewees: Onza cat (*Leopardus* sp.), Puma (*Puma concolor*), Jaguar, Common hog-nose skunk (*Conepatus chinga*), Greyheaded tayra (*Eira barbara*), Roadside hawks, Southern caracaras, Andean condors, hawks (*Accipiter* spp.), vultures and eagles. For the last three, we were not able to identify the species that the interviewees referred to. Of those 16 species, 11 preyed on chickens and chicks, three preyed both on poultry and livestock, while only two were considered to prey exclusively on livestock. Although the number of species that were considered poultry predators is higher than livestock predators, the number of reports of conflict due to this situation was lower for the former (34/115) compared to those generated by livestock loss (91/115). Of all conflictive species, the Andean condor and the Puma were considered the most conflictive species (X_2_ = 103.09, df = 6, *p* < 0.001) and obtained the highest conflict indexes (Table 2). For the rest of the raptor species evaluated no conflicts were reported.

The Occupation categories *teachers*, *livestock production*, *farming* and *others* presented higher conflict indices than *ornithological guides*, *outdoor occupations* and *park rangers* (Table 3). In particular, conflicts reported by workers from the former two occupations were centered on Andean condors and Pumas, while farmers’ reports were evenly distributed among the different species. In this way, 60% (24/40) of the interviewees that considered Andean condors as a conflictive species were involved in *livestock production* as their main source of subsistence (X^2^ = 10.9, df = 1, *p* < 0.001). Furthermore, 54.54% (24/44) of interviewees involved in *livestock production* and 80% (4/5) of *teachers* reported the existence of conflict with condors. Andean condor was the only species considered conflictive by people of all occupation categories.

Although only 12.07% (7/58) of the interviewees that reported that Andean condors were predators assured witnessing attacks, description of the preying mechanism used by condors were similar in most reports. Interviewees described that condors attack newly born calves by first pecking their tongue, eyes or anus while they are alive. Then they kept pecking the calves jumping on top of them and eventually throwing them to the ground, until they got to the intestines. On the other hand, 17.5% (7/40) mentioned that Andean condors are a problem only in the open “cerro” areas and not in the closed “monte” areas. 

Most interviewees that reported having conflicts with Jaguars and Pumas mentioned that they were able to differentiate the attacks of each species by their behavior. According to them, Pumas are associated with surplus killing (defined as the slaughtering of more than is required for immediate consumption [77]), and attack calves, hens, foal, lambs and young goats; while Jaguars were only accused of preying on one individual adult cow or calf per time (no surplus killing).

Most interviewees (85.21%, 98/115) said that they do not take actions in response to conflicts, while only seven reported that they took mitigation actions to avoid them, such as guarding livestock themselves or with herding dogs, or installing hen houses for poultry. Interviewees that took actions in retaliation for presumed predation (10/115) said they use a slingshot to scare off raptors (6/115) or they kill the problematic animal with firearms or toxic baits (10/115). Two interviewees declared to have hunted a condor and one to have hunted an undetermined eagle.

### 3.4. Relation of Socio-Demographic Variables With Social Perception of Conflict With Wildlife

The variables ethnic affiliation and place of residence were excluded from the GLM analysis, as their variance inflation factors (vif) were higher than 10, indicating collinearity. None of the socio-demographic variables were associated with the response variable conflict with wildlife reported by interviewees except for poultry rearing and livestock rearing, which are highly significant (Table 4). Rearing of both types of domestic animals was positively associated with the existence of HWC.

On the other hand, for the response variable *conflict with Andean condor* we included the explanatory variable *HWC with other species*. As this was the most conflictive species we evaluated if conflict with this species was correlated with conflict reports involving other species. Both *livestock rearing* and *HWC with other species* were significant variables in the Anova analysis and were positively associated with the existence of conflict with Andean condors (Table 5). For the response variable *conflict with Puma*, the explanatory variable *livestock rearing* was the only significant variable in the Anova analysis, being positively associated with the existence of conflict with Pumas (Table 6).

For the GLM analysis of the response variable *conflicts with Roadside hawk*, the social factor *occupation* was excluded due to convergence issues. *Poultry rearing* was the only significant variable in the Anova analysis (Table 7). Nonetheless, the coefficient estimate for this variable was not significant in the GLM analysis, showing a high standard error. All factors were non-significant for the models run for Jaguars, Southern caracaras and Eagles.

## 4. Discussion

Overall, our results suggest that in the Yungas of Argentina, human attitudes towards raptors tend to be neutral, except for eagles, for which some interviewees expressed positive attitudes, and for the Andean condor, for which a third of the interviewees expressed negative attitudes. The general neutrality of people towards raptors could be related to the fact that the local human population seems to have poor knowledge of the raptor community that surrounds them, except for some high profile or very common species, such as the Andean condor, the Southern caracara, the Roadside hawk and the Black-chested buzzard-eagle [45,68]. Nonetheless, although these species are well known and considered predators of domestic animals by local settlers, conflicts arise mostly around the Andean condor (ICos = 34.78), and less so around the Roadside hawk (ICos = 14.78), while for Southern caracara and “eagles” the conflict is almost nonexistent (ICos = 5.22). This contrasts with results from other areas such as the northern Patagonia in Argentina, where Southern caracara and Black-chested buzzard eagle were considered harmful by at least half of the interviewees, while the Andean condor was considered harmful only by 25.5% of the 51 farmers interviewed [21]. Differences amongst regions in livestock husbandry practices and the kind of livestock raised, and thus on the size of the animals raised, may influence which species are perceived as predators and/or have been recorded in predatory behaviors. In the Yungas socio-ecological system, conflict reports associated with eagles and Southern caracaras were related to the predation of poultry and small size livestock, such as lambs, young goats and piglets. These livestock species were raised by a small proportion of our interviewees in the study area, mostly for self-consumption or as an additional income source. Instead, most interviewees involved in livestock rearing raise cattle. This contrasts with other areas of Argentina, like Patagonia, where settlers with conflicts with raptors are mainly involved in extensive sheep farming [21]. Likewise, although Roadside hawks were commonly identified as chicken predators in the Yungas, few people declared actually having a problem and taking actions towards this HWI, while in a study describing and quantifying the poaching of wildlife in Brazil, this species was the only raptor reported to be poached by the 23 people that were interviewed and it was the second species with the most deaths [50]. In our study, interviewees reported poaching roadside hawks only when they were found preying on their poultry, while in the Silva de Lima study, almost half of the poached animals were killed to prevent attacks. The Crowned solitary eagle was not identified by interviewees, and thus no conflicts were reported in our study area; in almost all the rest of Argentina where the species occurs, cases of persecution were recorded [41]. This species is likely not present or occurs only rarely in the Yungas environments where we carried out the interviews.

Unlike previous studies carried out in Colombia in which at least half of the interviewees perceived the Black-and-chestnut eagle (BCE) as a harmful bird or reported losses of poultry due to this species [29,30,49], we did not record reports of conflict with the BCE. This may be associated with the fact that the species is extremely rare in Argentina and thus seems to be poorly known in our study area [68], while almost all rural settlers interviewed in Colombia recognized the species. Of the 63 BCE individuals that were shot or captured in Colombia 60% were killed in retaliation for poultry predation [30,49]. Surprisingly, although predation on poultry by BCE has also been recorded in the only nest monitored in Argentina [55], none of the interviewees living in close proximity of this nest knew the species, and poultry losses were attributed to other predators. Furthermore, these losses were not considered of relevance. The fact that these people do not recognize the BCE may be related to a dissociation between local settlers and their surrounding ecosystems due to socio-environmental changes in the past century. Changes in subsistence activities related to the environment possibly affected peoples’ knowledge on wildlife, including raptors [78,79]. Another possible explanation for the differences found in the Colombian studies [29,30,49] is that the particular socio-cultural context under study implies different appreciations of domestic animals, both in economic and cultural terms. Therefore, the importance attributed to the loss of certain animals may vary. For instance, although in our study the number of reports of species feeding on poultry (14 species) was higher than reports of species feeding on livestock (5 species), the number of interviewees that expressed conflict with poultry predators was lower compared to conflict reports for species that prey on livestock. Given that the proportion of interviewees that possess poultry and livestock was similar, these results suggest that in most cases poultry has a lower economic and/or cultural value, or at least a secondary value for local settlers of this region, in contrast with livestock, which is highly valued. The Yungas socio-ecological system is characterized by the historical and cultural practice of transhumance, a strongly marked mobile economy centered on livestock and shifting agriculture [66], therefore any damage to livestock is expected to be of great relevance among local settlers. This is in correspondence with *livestock rearing (past or present)* being the single most important and significant socio-demographic factor associated with conflict. Results of conflict indices are also aligned with this fact: species perceived or known as livestock predators had the highest values of conflict (Andean condor-ICos = 34.78, Puma- ICos = 28.67, Jaguar- ICos = 22.99). This could also explain why conflict with Andean condor was associated with the existence of conflict with other species, as most reports made by interviewees involved livestock as the main cause of conflict. In our study area, many people have kept a tight relationship with livestock raising even when they have other jobs as their main income source. For example, *teachers* was the occupation category with the highest score of conflict (OCi = 1.6), but 4 of 5 teachers interviewed were also involved in livestock rearing. This general use of livestock rearing as a second income source may have included noise in the analysis precluding the existence of a clear relationship between the interviewees’ occupation and the existence of conflict in general or in particular. People for whom *livestock production* was their main job were the second group expressing conflicts with wildlife (OCi = 1.36). However, given we did not assess the relationship between local settlers and their domestic animals, and we did not evaluate the extent and characteristics of each interviewee’s rearing practices, further research is needed to clarify how the cultural and economic importance of each type of domestic animal may interplay in the building of HWC. 

Livestock production is characterized by an intertwining of multiple considerations, including not only its economic importance, but also social and cultural relevance, health value and contribution to food security [80]. In the last decades, social and economic changes, associated with a shift in complementary agricultural temporal job offers, the construction of roads and migration of younger generations to larger towns, led to the degradation of husbandry practices in the Yungas with a reduction in livestock number and care, yet not in a complete abandonment of the practice [67,81]. This adaptation to the present socio-economic context implies that in many cases livestock ranges freely and unattended even during calving, and are only rarely checked by herders that are now mainly dedicated to other labor or occupations. Some local settlers acknowledged that the lack of appropriate husbandry practices facilitates the occurrence of conflictive situations: without the company and presence of herders, livestock remain exposed to predators, which increases the probability of predation [82]. At the same time, in this new context, deaths or losses related to other causes, such us diseases, may also be attributed to wild predators such as Pumas, Jaguars and Andean condors, that may have in fact consumed the remains of these animals in an opportunistic manner [21,50,83,84]. This may produce an increase in the perception of risk and reinforce negative attitudes towards predators [24], leading to further retaliation against carnivores. This includes the use of poisoned carcasses to control them, which affects a large number of non-target species [85] and is considered a serious threat for the natural populations of endangered species such as some vulture species worldwide [86].

Vulture-human relationships have changed through history under shifting socio-ecological scenarios [87]. Although historically this relationship was considered beneficial, coexistence in the present has been disrupted. Globally, the number of reports of attacks by species that are classified as exclusively scavengers are rising, and reports of conflict between vultures and humans due to livestock losses are widespread [88,89,90,91,92]. The Andean condor is no exception, as although it is considered an obligate scavenger by most of the scientific community, it is considered a livestock predator by rural communities [21,34,48]. Although at present there are scarce scientific records of Andean condors engaged in this predatory behavior, there is a large bulk of attack reports by local people collected by previous scientific studies [48,92] as well as by the press (valledelermahoy.com.ar/un-condor-es-colgado-en-un-arbol-por-un-productor-ganadero-video/) and filmed by civilians (e.g., https://www.youtube.com/watch?v=4ljE5BSQoD8, accessed on 11 May 2021). Close similarities were found among all the descriptions of condor behavior and strategies during the attacks to calves provided to us by different stakeholders, which were also very similar with reports recorded in other study areas [21,38,48] F.H.V. pers. comm. Nonetheless, researchers and conservationists still assume this species to be an obligate scavenger and therefore fail to manage the problem and to engage locals in conservation practices [92]. 

Studies carried out in Patagonia suggest that at least locally, the amount of damage produced by condors is not aligned with local settlers’ perception of damage levels [21]. However, damage exists and was recorded, and therefore acknowledging Andean condor predatory behavior and taking mitigation actions would probably help to reduce conflict in certain situations [12,92]. Furthermore, to fully comprehend and manage the specific human-condor conflict, it is necessary to consider that the damage perceived is associated with both tangible monetary costs and intangible costs. This is suggested in the study of Ballejo et al. [93], where most farmers that considered at least some scavenger birds as harmful lamb predators, also considered losses by scavengers negligible or acceptable. Intangible factors, such as fear, trust in the management agency, perceptions of control over the situation, beliefs and affection or dislike for the species, may be taking a main role in shaping this particular human-wildlife relationship [94]. These factors may be rooted in cultural views of local settlers, which conservation agents could be questioning when they intervened in HWI, exacerbating HWC through human-human conflict. Scientists, legislators and other related stakeholders usually pursue top-down approaches, such as imposed political resolutions and legal mandates [8], adopting a position in which the truth-telling power of the scientific method is put in direct opposition and above of how locals experience and explain human–wildlife relations [95]. Such is the case for the Andean condor, where failure to recognize the condor predatory capacity leads local settlers to mistrust scientists, questioning their neutrality when addressing conservation challenges. At the same time, this may be perceived as a persistence of historical power struggles and disputes over land ownership and the use of natural resources [8,23,96]. In our study area, the rural population historically has been characterized by inequality and imbalances of power, since local settlers were expelled from their territories for agriculture production and the creation and administration of protected areas [81,97] and therefore conservation agents may be associated with historical wounds and the persistence of past and present injustices [2]. 

In our study, Andean condors were considered more conflictive than Pumas and Jaguars, two feline species known as livestock predators for which HWC are well known and widely studied in different regions of South America [98,99,100]. Although it is possible that the conflict with these two felines is bigger than what we recorded in our study, as our interviews were not focused on mammals, interviewees that mentioned both Condor and Puma and/or Jaguar usually considered them equally conflictive. There is at least one previous study where Pumas and Condors were also considered as equally harmful in San Juan province (central Argentina), and several studies have shown that there is a general perception of the Andean condor as a livestock predator across different environments in Argentina [21,34,48]. Like in the case of the BCE, there are local or regional differences in the conflictive nature of the species across different areas, probably related to each particular socio-ecological context, including the vulnerability of the affected families or producers [24]. Therefore, further studies are needed to obtain a better understanding of the local characteristics of the HWC involving Andean condors that occur across the different socio-ecological systems where the species is present in Argentina. 

Reports of Condor persecution (and persecution of wildlife in general) were scarce in our study, although given the illegality of the activity, it is possible that some interviewees may have omitted this information. In any case, the increasing number of mass Condor poisonings, clearly highlights the need to fully address this conflict and in general the HWC with predators in the species distribution range. The widespread negative perception of the Andean condor as a livestock predator along with the strong belief that scientists mistrust and ignore locals’ knowledge and experiences suggest that true intercultural dialogue and transdisciplinary approaches with strong involvement of social scientists, and an honest inclusion of locals will be needed to improve this conflictive situation if we are to avoid the escalation of a conflict that could be a death sentence for a highly sensitive species like the Andean condor.

## 5. Conclusions

We assessed human-raptor conflict using an interdisciplinary approach in the study area comprising the Yungas ecoregion of Salta and Jujuy, Northwestern Argentina. This is the first assessment for this area, filling a knowledge gap and helping to identify top priority species and human focus groups involved in HWC. Conflicts were only concentrated in some high-profile raptor and mammal species, particularly those considered livestock/cattle predators, showing a general high tolerance towards species considered poultry predators. Even more, except for the Andean condor, local settlers showed little interest in and a high coexistence with most raptor species evaluated in our study. The BCE and the Andean condor case study highlight the importance of the particular relationships that have developed between predators, prey and people at certain regions. However, we cannot disesteem that other human-raptor conflicts could exist in other sites of the Yungas region, and further research is needed to fully comprehend the HWI and underlying HHC. 

On the other hand, although persecution of wildlife seems to be low in the study area, calls for conservation efforts are needed to address the existing conflict with the Andean condor. Our results indicated that local settlers strongly perceived this species as a livestock predator, contrary to what science reports. These contradictory perceptions between different stakeholders may risk attempts to address the problem. This could lead to a further increase of HHC, and an escalation of the problem, which could be detrimental for the Andean condor, and in general for biodiversity conservation in the region. To tackle this problem, conservation agents should remember that nature conservation is highly subjective and a value laden science, and that behind HWC often lies HHC related to differences in fundamental values, attitudes, goals and historical wounds among the various stakeholders [9]. In this context, to diminish HHC and increase trust and cooperation between stakeholders, local communities should have an equal status and ownership in the planning and evaluation of conservation strategies that integrate ecological, social, economic and political contexts within which all biodiversity and human-wildlife conflicts are embedded [6,7,101]. Only by means of a true intercultural dialogue and transdisciplinary approaches may the goal of biological (or better, biocultural) conservation be achieved.

## Figures and Tables

**Table 1:** Demographics from interviewees.

Variable	Category	*n*	Percentage
Gender	Man	87	75.65
	Woman	28	24.35
Age (years)	18–95		
Occupation	*Livestock production*	44	38.26
	*Outdoor occupation*	8	06.96
	*Farming*	16	13.91
	*Ornithological guide*	9	07.83
	*Others*	20	17.39
	*Parkranger*	13	11.30
	*Teachers*	5	04.35
Ethnic Affiliation	Non-indigenous people	84	73.04
	Indigenous people	31	26.96
Settlement type	City	10	08.70
	Rural town	74	64.35
	Ranch	31	26.95
Time of residence (years)	1–87		
Livestock rearing	Yes	77	66.96
	No	38	33.04
Poultry rearing	Yes	78	67.83
	No	37	32.17

**Table 2:** Number of interviewees that reported the existence of conflict and conflict indexes (ICos) for each species or group of species (i.e., eagles). Only conflicts with ICo ≥ 5% are included.

Species	Number of Conflict Reports	ICos(%)
Andean condor	40	34.78
Puma	33	28.70
Jaguar	20	22.99
Roadside hawk	17	14.78
Eagles	6	5.22
Southern Caracara	6	5.22

**Table 3:** Number of HWC reports gathered for each occupation category and conflict indices by occupation.

Occupation	Number of HWC Reports	OCi
* Teachers *	8	1.60
* Livestock production *	60	1.36
* Others *	23	1.15
* Farming *	18	1.13
* Park ranger *	8	0.62
* Ornithological guide *	4	0.44
* Outdoor occupation *	3	0.38

**Table 4:** Deviance analysis results showing the effect of each factor used in the binomial generalized linear model for the response variable wildlife conflict and their significance, performed with the Anova function of “car” package. Explanatory variables with *p*-values < 0.05 are in bold. Estimated coefficients and standard errors are shown for the significant categories (yes) of the significant social factors.

Explanatory Variables	Chi^2^	Degrees of Freedom	*p*-Value	Estimate	Standard Error
Gender	0.596	1	0.440		
Age	0.202	1	0.653		
Time of residence	1.024	1	0.312		
Occupation	10.357	6	0.110		
Settlement type	0.111	2	0.946		
Poultry rearing	5.829	1	**0.016**	**0.727**	**0.315**
Livestock rearing	8.435	1	**0.004**	**0.860**	**0.306**

**Table 5:** Deviance analysis results showing the effect of each factor used in the binomial generalized linear model for the response variable conflicts with Andean condors and their significance, performed with the Anova function of “car” package. Explanatory variables with *p*-values <0.05 are in bold. Estimated coefficients and standard errors are shown for the significant categories (yes) of the significant social factors.

Explanatory Variables	Chi^2^	Degrees of Freedom	*p*-Value	Estimate	Standard Error
Gender	0.001	1	0.988		
Age	0.365	1	0.546		
Time of residence	0.013	1	0.909		
Occupation	8.077	6	0.232		
Settlement type	5.727	2	0.057		
Poultry rearing	1.635	1	0.201		
Livestock rearing	7.158	1	**0.007**	**2.313**	**0.985**
HWC with other species	4.852	1	**0.028**	**1.153**	**0.535**

**Table 6:** Deviance analysis results showing the effect of each factor used in the binomial generalized linear model for the response variable conflicts with Pumas and their significance, performed with the Anova function of “car” package. Explanatory variables with *p*-values < 0.05 are in bold. The estimated coefficient and standard error are shown for the significant category (yes) of the significant social factor.

Explanatory Variables	Chi^2^	Degrees of Freedom	*p*-Value	Estimate	Standard Error
Gender	0.336	1	0.562		
Age	0.459	1	0.498		
Time of residence	3.285	1	0.070		
Occupation	9.623	6	0.141		
Settlement type	4.945	2	0.084		
Poultry rearing	0.044	1	0.833		
Livestock rearing	9.714	1	**0.002**	**2.381**	**0.855**

**Table 7:** Deviance analysis results showing the effect of each factor used in the binomial generalized linear model for the response variable conflicts with Roadside Hawks and their significance, performed with the Anova function of “car” package. Explanatory variables with *p*-values <0.05 are in bold. The estimated coefficient and standard error are shown for the significant category (yes) of the significant social factor.

Explanatory Variables	Chi^2^	Degrees of Freedom	*p*-Value	Estimate	Standard Error
Gender	2.286	1	0.131		
Age	1.951	1	0.163		
Time of residence	1.610	1	0.205		
Settlement type	0.859	2	0.651		
Poultry rearing	19.352	1	**<0.001**	**19.487**	**1657.346**
Livestock rearing	1.0482	1	0.306		

## Data Availability

The data that support the findings of this study are available from the corresponding author upon reasonable request.

## Supplementary Materials

The following are available online at https://www.mdpi.com/article/10.3390/ani11051428/s1. Table S1: Predatory and scavenger raptors present in the Argentine Yungas.
