# Supplementary material for: Human-Wildlife Conflicts in the Southern Yungas: What Role do Raptors Play for Local Settlers?
Source: Animals (Basel). 2021 May 17;11(5):1428. doi: 10.3390/ani11051428 (PMC8156693; doi:10.3390/ani11051428)
Supplement: Supplementary file 1 [file animals-11-01428-s001.zip › animals-1172239-supplementary.pdf]

Supplementary

Table S1: Predatory and scavenger raptors present in the Argentine Yungas

Name	Species	Conservation status		Type	Registered by our work group	Recognized by interviewees	Target group
		UICN	Argentina				
Andean condor	*Vultur gryphus*	**VU**	**Vulnerable**	Resident	x	x	x
King vulture	*Sarcoramphus papa*	LC	Not Threatened	Resident	x	x	
Turkey vulture	*Cathartes aura*	LC	Not Threatened	Resident	x	x	
Lesser yellow-headed vulture	*Cathartes burrovianus*	LC	Not Threatened	Resident			
Black vulture	*Coragyps atratus*	LC	Not Threatened	Resident	x	x	
Black-chested buzzard eagle	*Geranoaetus melanoleucus*	LC	Not Threatened	Resident	x	x	x
Black-and-white hawk-eagle	*Spizastur melanoleucus*	LC	**Vulnerable**	Resident		x	x
Short-tailed Hawk	*Buteo brachyurus*	LC	Not Threatened	Resident	x		
Swallow-tailed kite	*Elanoides forficatus*	LC	Not Threatened	Migrant	x	x	
White-tailed kite	*Elanus leucurus*	LC	Not Threatened	Resident	x		
Pearl kite	*Gampsonyx swainsonii*	LC	Not Threatened	Resident			
Mississippi kite	*Ictinia mississippiensis*	LC	Not Threatened	Migrant			
Plumbeous kite	*Ictinia plumbea*	LC	Not Threatened	Migrant	x		
Roadside hawk	*Rupornis magnirostris*	LC	Not Threatened	Resident	x	x	x
Great black hawk	*Buteogallus urubitinga*	LC	Not Threatened	Resident	x	x	x
Bay-winged hawk	*Parabuteo unicinctus*	LC	Not Threatened	Resident			
Snail kite	*Rostrhamus sociabilis*	LC	Not Threatened	Resident			
Long-winged harrier	*Circus buffoni*	LC	Not Threatened	Resident			
Cinereous harrier	*Circus cinereus*	LC	Not Threatened	Resident	x		
Bicolored hawk	*Accipiter bicolor*	LC	Not Threatened	Resident	x	x	
Sharp-shinned hawk	*Accipiter striatus*	LC	Not Threatened	Resident	x		
White-rumped hawk	*Buteo leucorrhous*	LC	**Endangered**	Resident	x	x	
Zone-tailed hawk	*Buteo Albonotatus*	LC	Not Threatened	Resident			
Hook-billed kite	*Chondrohierax uncinatus*	LC	Not Threatened	Migrant	x		
Gray hawk	*Asturina nitida*	LC	**Vulnerable**	Resident			
Rufous-thighed kite	*Harpagus diodon*	LC	Not Threatened	Resident			
White-tailed hawk	*Geranoaetus albicaudatus*	LC	Not Threatened	Resident	x		
Swainson's hawk	*Buteo swainsoni*	LC	**Vulnerable**	Migrant			
Red-backed hawk	*Geranoaetus polyosoma*	LC	Not Threatened	Resident	x	x	x
Crane hawk	*Geranospiza caerulescens*	LC	Not Threatened	Resident	x		
Solitary eagle	*Harpyhaliaetus solitarius*	**NT**	**Endangered**	Resident	x	x	
American harpy eagle	*Harpia harpyja*	**NT**	**Critically endangered**	Resident		x	x
Ornate hawk-eagle	*Spizaetus ornatus*	**NT**	**Endangered**	Resident		x	x
Black-collared hawk	*Busarellus nigricollis*	LC	Not Threatened	Resident			
Savanna hawk	*Buteogallus meridionalis*	LC	Not Threatened	Resident	x	x	
Crowned eagle	*Buteogallus coronatus*	**EN**	**Endangered**	Resident		x	x

Table S1: Continued.

Name	Species	Conservation status		Type	Registered by our work group	Recognized by interviewees	Target group
		IUCN	Argentina				
Black-and-chesnut eagle	*Spizaetus isidori*	**NT**	**Endangered**	Resident	x	x	x
Southern caracara	*Caracara plancus*	LC	Not Threatened	Resident	x	x	x
Chimango caracara	*Phalcoboenus chimango*	LC	Not Threatened	Resident	x		
Collared forest-falcon	*Micrastur semitorquatus*	LC	**Vulnerable**	Resident		x	
Barred forest-falcon	*Micrastur ruficollis*	LC	Not Threatened	Resident	x		
Spot-winged falconet	*Spiziapteryx circumcinctus*	LC	**Vulnerable**	Resident			
Peregrine falcon	*Falco peregrinus*	LC	Not Threatened	Resident	x	x	
Aplomado falcon	*Falco femoralis*	LC	Not Threatened	Resident	x		
Orange-breasted falcon	*Falco deiroleucus*	**NT**	**Endangered**	Resident	x	x	
Bat falcon	*Falco rufigularis*	LC	Not Threatened	Resident	x		
American kestrel	*Falco sparverius*	LC	Not Threatened	Resident	x		

Supplementary

HUMAN-RAPTORS INTERACTION SURVEY

Questions and themes included in this survey are a guide of the topics and information that should be addressed by the interviewer. Surveys adjusted to the interviews development, therefore questions were not addressed in the same way or the same order on each interview.

Personal information of interviewees

Interviewee Name: _______________
Age: _______________
Gender: _______________
Ethnic affiliation: _______________
Place of origin (place where it was born and/or raised): _______________
How long has it been since you settle in the area?
Settlement type: _______________
Sites where you have lived: _______________
Activities/occupations/Actual or past expertise:
Are you or have you been involved in livestock rearing? Which species? Which husbandry practice do you apply?
Are you or have you been involved in poultry rearing? Which husbandry practice do you apply?
Field visits (With what purpose? Do you go alone, with a partner or in group? How long does each visit last? Where do you go? Why?)
Hunting history: _______________
Description of the area (have there been changes in the area since you lived here? What was it like before?)
Other biographic information.

Knowledge, perceptions and attitudes towards raptors

Description of the raptors known.
Morphological and behavioral knowledge of raptors known.
Raptors identification in the visual supplementary material.
How are they call?
What do raptors eat? Prey´s list.
Perceptions and attitudes towards raptors.

Human-wildlife Conflict

Do you have conflicts with a particular animal? Why? Are they harmful for crops? Are they harmful for domestic animals?
Conflicts with raptors.
List the animals that prey on livestock (cattle, sheeps, goats, pigs and horses).
List the animals that prey on poultry.
How do you know which is the conflictive animal? Does it leave marks and/or footprints? Does it have a particular behavior? Have you seen it? Has someone told you about it?
What is the importance of domestic animals lost?
Response towards conflict. Do you take prevention measurements? What actions do you take to stop or prevent the conflict?
Persecution of conflictive species.
